# Osteogenesis imperfecta in Brazilian patients

**DOI:** 10.1590/1678-4685-GMB-2018-0043

**Published:** 2019-08-15

**Authors:** Maira Trancozo, Marcos V.D. Moraes, Dalila A. Silva, Jéssica A.M. Soares, Clara Barbirato, Márcio G. Almeida, Lígia R. Santos, Maria R. G. O. Rebouças, Akel N. Akel, Valentim Sipolatti, Vanda R. R. Nunes, Flavia I. V. Errera, Meire Aguena, Maria R. Passos-Bueno, Flavia de Paula

**Affiliations:** 1 Núcleo de Genética Humana e Molecular, Departamento de Ciências Biológicas, Centro de Ciências Humanas e Naturais, Universidade Federal do Espírito Santo, Vitória, ES, Brazil; 2 Programa de Pós-Graduação em Biotecnologia, Centro de Ciências da Saúde, Universidade Federal do Espírito Santo, Vitória, ES, Brazil; 3 Hospital Estadual Infantil Nossa Senhora da Glória, Vitória, ES, Brazil; 4 Escola Superior de Ciências da Santa Casa de Misericórdia de Vitória, Vitória, ES, Brazil; 5 Instituto de Biociências, Universidade de São Paulo, São Paulo, SP, Brazil

**Keywords:** NGS gene panel, *COL1A1/COL1A2* genes, *FKBP10* gene, *P3H1* gene, *IFITM5* gene

## Abstract

Osteogenesis Imperfecta (OI) is a heterogeneous genetic disorder characterized by bone fragility and fracture. Mutations in 20 distinct genes can cause OI, and therefore, the genetic diagnosis of OI is frequently difficult to obtain because of the great number of genes that can be related with this disease. Studies that report the most frequently mutated genes in OI patients can help to improve molecular strategies for diagnosis of the disease. In order to characterize the mutation profile of OI in Brazilian patients, we analyzed 30 unrelated patients through SSCP screening, NGS gene panel, and/or Sanger sequencing for the 11 most frequently mutated genes in the database of mutations, including *COL1A1*, *COL1A2, P3H1*, *CRTAP*, *PPIB*, *SERPINH1*, *SERPINF1*, *FKBP10, SP7, WNT1* and *IFITM5*. Disease-causing variants were identified in *COL1A1*, *COL1A2*, *FKBP10, P3H1,* and *IFITM5*. A total of 28 distinct mutations were identified, including seven novel changes. Our data show that the analysis of these five genes is able to detect at least 95% of causative mutations in OI disorder from Brazilian population. However, it has to be taken into considerations that distinct populations can have different frequencies of disease-causing variants. Hence, it is important to replicate this study in other groups.

## Introduction

Osteogenesis Imperfecta (OI) is a heterogeneous group of connective tissue syndromes characterized by abnormal bone fragility that leads to fractures and skeletal deformities. The prevalence of OI is estimated to be 1/15,000 (Folkestad *et al.*, 2016). Because of its wide clinical variability, patients can also develop short stature, dentinogenesis imperfecta, blue sclera and hearing loss (van Dijk *et al.*, 2010). The phenotypic spectrum of OI may overlap with other skeletal diseases, which makes the establishment of a precise diagnosis based on clinical, radiological and genetic investigations extremely difficult. Based on clinical diagnosis, including pre- and postnatal severity of bone fragility, the traditional classification distinguishes four phenotypic groups (OI types I to IV). OI type I is the mildest phenotype, OI type II is lethal in the neonatal period, OI type III is the most severe form compatible with postnatal survival, and OI type IV represents a moderate form of severity (Sillence *et al.*, 1979). However, an expanded classification has been suggested based on the phenotype and the mutated gene (Marini *et al.*, 2017).

OI-like syndromes with a dominant or recessive pattern of inheritance can be associated with at least twenty genes (Dalgleish, 1997,^1998^; Kang *et al.*, 2017) . A large frequency of OI cases that segregate in an autosomal dominant pattern are due to heterozygous mutations in the structural genes coding for the two procollagen chains, *COL1A1* (OMIM 120150) and *COL1A2* (OMIM 120160), that form type I collagen structure, the main protein of bone, tendons and cartilage (Van Dijk *et al.*, 2010). In addition, mutations in *IFITM5* (OMIM 614757), the gene encoding BRIL, a transmembrane protein enriched in osteoblasts during mineralization, are less frequent but are also related (Cho *et al.*, 2012; Semler *et al.*, 2012; Hanagata, 2016). Mutations in the *P4HB* gene (OMIM 176790) were also reported causing OI with an autosomal dominant inheritance (Rauch *et al.*, 2015).

Those individuals who do not have a mutation in one of the structural collagen genes might carry mutations in genes with an autosomal recessive pattern. The number of genes discovered that may lead to recessive phenotypes have increased dramatically (Kang *et al.*, 2017; Marini *et al.*, 2017) . Among them are the following: *CRTAP* (OMIM 605497), *P3H1* (OMIM 610339), *PPIB* (OMIM 123841), *SERPINH1* (OMIM 600943), *FKBP10* (OMIM 607063), *SERPINF1* (OMIM 172860), *SP7/OSX* (OMIM 606633), *PLOD2* (OMIM 609220), *BMP1* (OMIM 614856), *TMEM38B* (OMIM 615066), *WNT1* (OMIM 615220), *CREB3L1* (OMIM 616229), *SPARC* (OMIM 616507), *MBTPS2* (OMIM 300294), *SEC24D (OMIM 607186)*. Mutations in these genes can cause abnormal collagen post-translational modification, collagen processing and crosslinking modification, bone mineralization, or osteoblast differentiation and function (Marini *et al.*, 2017). Recently, an X-linked gene, the *PLS3* (OMIM 300131), was included in the OI variant database (Dalgleish, 1997,^1998^).

According to the best practice guidelines for laboratory diagnosis of OI, Sanger sequencing of relevant genes is the gold standard (van Dijk *et al.*, 2012). Given the large number of suspected genes linked to OI and its phenotypic heterogeneity, Sanger sequencing of various genes might increase the sensitivity of molecular diagnosis of a disease. However, the procedure is not only expensive but also laborious and very time-consuming. The emergence of new technologies for high-throughput capture and the possibility of including all causative-OI genes in a sequencing panel has made next-generation sequencing (NGS) one of the most promising techniques for molecular diagnostic purposes (Sule *et al.*, 2013; Árvai *et al.*, 2016).

Herein, we present the results of the analysis of 30 individuals with typical OI phenotype from the Brazilian population through single strand conformation polymorphism (SSCP) screening, NGS gene panel, and Sanger sequencing for the *COL1A1*, *COL1A2, P3H1*, *CRTAP*, *PPIB*, *SERPINH1*, *SERPINF1*, *FKBP10, SP7, WNT1* and *IFITM5* genes.

## Material and Methods

### Subjects

A total of 30 patients with clinical and radiological diagnoses of OI were included in this study. All patients were selected from Hospital Estadual Infantil Nossa Senhora da Glória (HINSG), Vitória, ES, in southeastern Brazil, with approval by its Ethics Committee. In most of the cases we could not collect DNA samples from other family members. The participants of this study were recruited from 2006 to 2012. All participants gave informed consent to participate in this study. Fifteen patients previously described by our group (Barbirato *et al.*, 2009, 2015, 2016; Moraes *et al.*, 2012) were re-analyzed in this study.

### Mutational analysis

The Osteogenesis Imperfecta Variant Database contains a compilation of genetic variants from 20 OI-related genes (Dalgleish, 1997, 1998). In the present work, we selected the *COL1A1*, *COL1A2, P3H1*, *CRTAP*, *PPIB*, *SERPINH1*, *SERPINF1*, *FKBP10, SP7, WNT1* and *IFITM5* genes. We chose these as they were reported as being the most prevalent with causative-OI mutations (Dalgleish, 1997, 1998).

DNA samples were collected from peripheral blood and extracted using the Miller *et al.* (1988) protocol. PCR of exons and exon/intron boundaries was performed in genomic DNA followed by single strand conformation polymorphism (SSCP) screening on polyacrylamide gel and Sanger sequencing of abnormal fragments for the analysis of the *COL1A1*, *COL1A2, P3H1*, *CRTAP*, *PPIB*, *SERPINH1*, *SERPINF1* and *FKBP10* genes until 2013. As the NGS gene panel can detect genetic variations with higher sensitivity than SSCP screening, we re-analyzed patients for whom the molecular diagnosis was not conclusive by May 2015 through an NGS panel that contained the *COL1A1*, *COL1A2, P3H1*, *CRTAP*, *PPIB*, *SERPINH1*, *FKBP10* and *SP7* genes. After July 2015, patients without conclusive molecular results were analyzed through direct Sanger sequencing for the *WNT1* and *IFITM5* genes.

For SSCP screening, all exons of analyzed genes and their flanking regions were screened using 5-7% acrylamide gels and the commercial version MDE^®^ Mutation Detection Enhancement Gel (Lonza Group Ltd., USA) to improve sensitivity. Fragments with abnormal patterns on SSCP gels were analyzed after silver staining, and Sanger sequencing was performed in an ABI PRISM® 3100 Genetic Analyzer (Applied Biosystems, USA). The primers used for the *COL1A1* gene were previously described by Körkkö *et al.* (1998); those used for *P3H1*, *CRTAP* and *PPIB* were reported by Barbirato *et al.* (2015). The primers for *COL1A2*, *SERPINF1*, *SERPINH1*, *FKBP10, WNT1* and *IFITM5* are described in the Tables S1    -S6.

For the NGS gene panel, we used a customized panel that analyzed segments of exons for the *COL1A1*, *COL1A2*, *P3H1*, *CRTAP*, *PPIB*, *SERPINH1*, *FKBP10* and *SP7* genes. This analysis was performed with a NEXTERA kit (Illumina, USA), which was used to prepare the sample library and to capture genes. A total of 10 ng DNA/sample was used for the target enrichment step. For quantification of the samples and to verify the length of the library, we used the High Sensitivity DNA kit in an Agilent 2100 Bioanalyzer (Agilent Technologies, USA). Quantitative PCR was performed by means of the KAPA Library Quantification Kit in a Real Time LightCycler^®^ System (Roche, DE). The captured libraries were sequenced with a MiSeq Sequencer (Illumina, USA).

NGS sequence reads were aligned to the human reference genome (hg19, GRCh37) with the Burrows-Wheeler Aligner (BWA, version 0.6.1) (Li and Durbin, 2009). To verify single-nucleotide variant (SNV) substitutions and small indels (INDELs), variants were called with SAM tools (version 0.1.18), Picard tools (version 1.60) and Genome Analysis Toolkit (GATK, version 1.5.21) (Li *et al.*, 2009; McKenna *et al.*, 2010). Genotypes were called at all positions with high-quality sequence bases and filtered to retain SNVs and insertion-deletions with Phred-like quality scores of at least 20.

The pipeline used in NGS gene panel for the analysis of the variants used the following filters: minor allele frequency in 1000 genomes < 0.001 (MAF_1000G < 0.001); eliminated variants 3 annotated as downstream/ upstream/ intergenic/ within non-coding genes; not in dbSNP; minimum coverage 30 reads (DP≥30) and, predicted as pathogenic by more than one prediction program (Shift +Polyphen).

Sanger sequencing validated all mutations identified by NGS gene panel and SSCP screening.

## Results

This study comprised a total of 30 unrelated OI patients (16 males, 14 females), based on clinical and radiological diagnosis. A severe phenotype was observed in 37% of the patients (11/30), a moderate in 23% (7/30), and a mild one in 40% (12/30) of the total. Lethal OI cases were not included in this work. Isolated OI patients accounted for 67% (20/30) of the sample. Additionally, positive familial history was reported by 30% (9/30) of the patients. There were affected members in more than one generation according to the autosomal dominant pattern of inheritance in eight distinct families. We observed one family (P.26) that only reported affected siblings, suggesting an autosomal recessive pattern. The assessment of familial history was not available for patient P.11. Only one patient (P.2) reported consanguinity of her parents.

Disease-causing variants were identified in 97% (29/30) of the study patients. The initial SSCP screening and Sanger sequencing allowed the detection of mutations in 11 patients (seven mutations in the *COL1A1* gene, one change in the *COL1A2* gene, two genetic variants in the *P3H1* gene, and one mutation in the *FKBP10* gene).

In the subsequent analysis, the NGS gene panel was performed in 20 patients, including 18 patients for whom mutations were not identified and in two whose the results were not conclusive in the previous analysis. The NGS gene panel allowed the identification of mutations in 17 patients (seven pathogenic changes in the *COL1A1* gene, eight mutations in the *COL1A2* gene, pathogenic variants in two patients in *FKBP10* gene). The NGS gene panel also allowed the confirmation of a likely pathogenic *P3H1* gene variant previously detected by SSCP in heterozygosity in patient P.26. This gene was related to a recessive OI pattern, but the second mutation in this patient was not identified, neither by SSCP nor by the NGS gene panel. Sanger sequencing for the *IFITM5* gene identified a mutation in one of the patients. The causative OI mutation was not detected in patient P.30.

Overall, a total of 28 distinct mutations were identified, including seven novel changes. Two variants were reported more than once in distinct families. The remaining 26 genetic variants were unique. In total, 14 mutations were found in the *COL1A1* gene, nine in the *COL1A2* gene, and one in the *IFITM5* gene. Four mutations were detected in the *FKBP10* gene. Two other unrelated patients carry mutations in the *P3H1* gene. No mutations were found in the *CRTAP, PPIB, SERPINF1, SERPINH1, WNT1* or *SP7* genes in the studied sample. The main results are listed in Table 1.

**Table 1:** Genetic variations found in Osteogenesis Imperfecta patients.

Patient	Gene	Exon/Intron	Mutation (cDNA)	Mutation (protein)	Mutation type	Zygosity	OI phenotype	Familial	Novel	Mutation detection technique	OI type
P.1	*COL1A1*	Exon 8	c.608G > T	p.Gly203Val	Missense	Heterozygous	severe	No	No	NGS	III
P.2	*COL1A1*	Exon 13	c.887G > T	p.Gly296Val	Missense	Heterozygous	mild	Yes	Yes	NGS	I
P.3	*COL1A1*	Intron 16	c.1056+1G > A	Splice Site	Splicing	Heterozygous	moderate	No	No	SSCP	IV
P.4	*COL1A1*	Exon 17	c.1138G > T	p.Gly380Cys	Missense	Heterozygous	mild	Yes	No	SSCP	I
P.5	*COL1A1*	Exon 18	c.1165G > T	p.Gly389Cys	Missense	Heterozygous	moderate	No	No	NGS	IV
P.6	*COL1A1*	Intron 27	c.1875+1G > C	Splice Site	Splicing	Heterozygous	severe	No	No	SSCP	III
P.7	*COL1A1*	Exon 32	c.2165G > C	p.Gly722Ala	Missense	Heterozygous	mild	No	Yes	NGS	I
P.8	*COL1A1*	Exon 37	c.2523delT	p.(Gly842Alafs*266)	Frameshift	Heterozygous	mild	Yes	No	NGS	I
P.9	*COL1A1*	Intron 37	c.2559+1G > A	Splice Site	Splicing	Heterozygous	mild	Yes	No	SSCP	I
P.10	*COL1A1*	Exon 40	c.2750delG	p.(Gly917Aspfs*191)	Frameshift	Heterozygous	severe	Yes	Yes	SSCP	III
P.11	*COL1A1*	Exon 44	c.3162delT	p.(Gly1055Alafs*53)	Frameshift	Heterozygous	mild	N/A	No	NGS	I
P.12	*COL1A1*	Exon 45	c.3235G > A	p.Gly1079Ser	Missense	Heterozygous	mild	Yes	No	SSCP	I
P.13	*COL1A1*	Exon 45	c.3235G > A	p.Gly1079Ser	Missense	Heterozygous	mild	Yes	No	NGS	I
P.14	*COL1A1*	Exon 45	c.3239delC	p.(Pro1080Leufs*28)	Frameshift	Heterozygous	severe	No	Yes	SSCP	III
P.15	*COL1A2*	Intron 14	c.693+2T > C	Splice Site	Splicing	Heterozygous	moderate	No	No	NGS	IV
P.16	*COL1A2*	Exon 16	c.739G > C	p.Gly247Arg	Missense	Heterozygous	moderate	No	No	SSCP	IV
P.17	*COL1A2*	Exon 34	c.2027G > A	p.Gly676Asp	Missense	Heterozygous	mild	No	No	NGS	I
P.18	*COL1A2*	Exon 38	c.2314G > A	p.Gly772Ser	Missense	Heterozygous	mild	No	No	NGS	I
P.19	*COL1A2*	Exon 38	c.2314G > A	p.Gly772Ser	Missense	Heterozygous	mild	Yes	No	NGS	I
P.20	*COL1A2*	Intron 40	c.2565+1G > A	Splice Site	Splicing	Heterozygous	moderate	No	No	NGS	IV
P.21	*COL1A2*	Exon 40	c.2458G > A	p.Gly820Ser	Missense	Heterozygous	severe	No	No	NGS	III
P.22	*COL1A2*	Exon 49	c.3278G > A	p.Gly1093Asp	Missense	Heterozygous	severe	No	No	NGS	III
P.23	*COL1A2*	Exon 49	c.3296G > C	p.Gly1099Ala	Missense	Heterozygous	severe	No	Yes	NGS	III
P.24	*IFITM5*	5`UTR	c.-14C > T	p.Met1ext-5	Start Codon	Heterozygous	severe	No	No	Sanger S	V
P.25	*P3H1*	Intron 5	c.1080+1G > T	Splice Site	Splicing	Homozygous	severe	No	No	SSCP	VIII
P.26	*P3H1*	Exon 14	c.2024G > T	p.Trp675Leu	Missense	Heterozygous	severe	Yes	No	SSCP/NGS	VIII
P.27	*FKBP10*	Exon 1	c.21dupC	p.Ser8Glnfs*67	Frameshift	Homozygous	moderate	No	No	NGS	XI
P.28	*FKBP10*	Exon 1	c.179A > C	p.Gln60Pro	Missense	Heterozygous	moderate	No	Yes	NGS	XI
		Intron 6	c.1063+2T > C	Splice Site	Splicing	Heterozygous					
P.29	*FKBP10*	Exon 5	c.831dupC	p.Gly278Argfs*95	Frameshift	Homozygous	severe	No	No	SSCP	XI
P.30	-	-	-	-	-	-	mild	No	-	-	-

N/A: Not available; NGS: next-generation sequencing gene panel; SSCP: single strand conformation polymorphism; Sanger S: Sanger sequencing

We also re-analyzed through NGS gene panel and Sanger sequencing the status of seven genetic variants: i) the c.1812C > T (p.Pro604=) synonymous variant in the *P3H1* gene; ii) the c.1087A > G (p.Lys363Glu) missense change in the *P3H1* gene; iii) the c.558A > G (p.Ala186=) change in the *CRTAP* gene; the two following changes present in the *FKBP10* gene: iv) the c.590A > G (p.Lys197Arg) missense change; and v) c.1546G > A (p.Leu516Phe) variant; and the two following changes in the *SERPINF1* gene: vi) the c.18A > G (p.Leu6=) synonymous variation, and vii) c.21C > A (p.Leu7=) change, previously reported by our group (Barbirato *et al.*, 2015, 2016). These variations were found in patients P.1, P.10, P.11, P.24, and P.27 who carry pathogenic known mutations (Table 1). The identification of these known pathogenic mutations in patients who carry these genetic variants suggests that these changes are rare non-pathogenic variants.

## Discussion

In our study, we analyzed 30 unrelated OI patients for the *COL1A1*, *COL1A2, P3H1*, *CRTAP*, *PPIB*, *SERPINH1*, *SERPINF1*, *FKBP10, SP7, WNT1* and *IFITM5* genes through SSCP screening, NGS gene panel and Sanger sequencing. We identified pathogenic mutations in 97% of the sample, including seven novel changes. Eighty per cent of causative OI mutations were detected in genes related to autosomal dominant OI (*COL1A1*, *COL1A2* and *IFITM5* genes) and 17% in genes involved in autosomal recessive OI forms (*P3H1* or *FKBP10* genes). Because there are different genes related to OI, and there is a lack of hotspots of mutations in most populations, knowledge about the primarily mutated genes that cause OI in specific populations can improve the strategies of genetic diagnosis for this disease.

In the present work, the study of the *COL1A1*, *COL1A2, P3H1*, *FKBP10* and *IFITM5* genes, selected from among 20 other distinct genes that can cause OI, allowed the detection of genetic changes in at least 95% of our subjects, providing supporting to the hypothesis that these genes contain most of the mutations found among OI patients. We failed to identify mutations in one patient. This likely occurred because this patient may carry pathogenic changes in a gene that was not analyzed in this study, or because the pathogenic variant is in a regulatory region that was not studied. When a causative OI mutation cannot be identified among the many genes studied, other genes must be analyzed to define the molecular diagnosis.

Bardai, *et al.* (2016), in a study from Canada that enrolled 598 OI individuals, proposed the division of patients into groups according to their phenotype to improve the identification of causative-OI mutations. In our sample, when we divided the patients using this parameter, all patients with a mild spectrum, and in whom the mutation was identified, showed mutations in the *COL1A1/COL1A2* genes. Bardai *et al.* (2016), also showed that 77% of patients with a moderate/severe spectrum carry mutations in the *COL1A1/COL1A2* genes, 9% of the patients share genetic changes in the *IFITM5* gene, and 12% of the patients carry mutations in genes related to a recessive OI pattern. The results of Bardai *et al.* (2016) suggest that the pathogenic changes are found mainly in *SERPINF1*, *CRTAP*, *P3H1*, *WNT1* and *FKBP10* genes among those related with recessive OI pattern. In our sample, we identified pathogenic changes related with recessive pattern only in *P3H1* and *FKBP10* genes.

The majority of causative-OI mutations identified were in the *COL1A1* gene, representing 47% (14/30) of all variants detected in this study, followed by 30% (9/30) in the *COL1A2* gene, 10% (3/30) in the *FKBP10* gene, 7% in the *P3H1* gene (2/30), and 3% (1/30) in the *IFITM5* gene. As reported in several works, 50% or more of the OI patients carry changes in the *COL1A1* gene, followed by the *COL1A2* gene (Zhang *et al.*, 2012; Lindahl *et al.*, 2015; Ho *et al.*, 2016). These works also described that most of the pathogenic variants in the *COL1A1/COL1A2* genes are glycine substitutions. As expected, approximately 60% of the *COL1A1/COL1A2* mutations identified in our study were glycine substitutions, while 40% of the patients carry other types of genetic variations, including mainly changes to splice sites and frameshift mutations.

Genes involved with recessive OI forms of inheritance that were previously reported by our group (Barbirato *et al.*, 2015, 2016) were re-analyzed in this study. SSCP screening allowed to find the *P3H1* c.2024G > T (p.Trp675Leu) change in heterozygous state in patient P.26. The NGS gene panel confirmed the presence of this variant in heterozygosity. Mutations in the *P3H1* gene cause OI type VIII. In patient P.26, no other genetic change was found in any of the studied genes. The second pathogenic change in this patient may be localized in a non-coding region of the *P3H1* gene, which was not analyzed in this work. However, as we did not find a second change, we cannot exclude the fact that the causative-OI mutation can be in another *locus*.

The use of the NGS gene panel allowed the identification of two variants in patient P.28, these being the c.179A > C (p.Gln60Pro) missense change (exon 1) and the c.1063+2T > C splicing site alteration (intron 6) in the *FKBP10* gene. Another change identified in the same gene by NGS methodology was the c.21dupC homozygous mutation detected in patient P.27. These three distinct mutations in the *FKBP10* gene are predicted to be pathogenic changes, according to *in silico* tools of mutation prediction present in the analysis of the NGS sequences. Mutations in the *FKBP10* gene cause OI type XI.

The *FKBP10* c.831dupC frameshift change and the *P3H1* c.1080+1G > T mutation, previously identified by our group (Barbirato *et al.*, 2015, 2016), are very well described in the literature (Fratzl-Zelman *et al.*, 2016; Caparros-Martin *et al.*, 2017). Therefore, they were re-analyzed only by Sanger sequencing. These homozygous changes were confirmed in patients P.29 and P.25, respectively. The *P3H1* c.1080+1G > T change has a carrier frequency of approximately 1/240 in the African American population. This mutation in homozygous state usually results in a perinatal lethal form of OI (Cabral *et al.*, 2007). In some populations, the carrier frequency of this allele is relatively high. As the carrier frequency in a population increases, the frequency of infants with recessively inherited OI due to homozygosity for one allele increases, as demonstrated by the homozygosity for *P3H1* c. 1080+1G > T among West Africans and the presence of founder mutations in other distinct geographic endogamous groups (Pepin *et al.*, 2013). In our sample, this change was observed only once among the 30 unrelated OI patients. The number of patients studied for this rare change in our work is too small to infer their approximate frequency in our population.

The c.-14C > T change identified in one patient in our sample is a recurrent mutation in the *IFITM5* gene described in the literature (Liu *et al.*, 2016, Guan *et al.*, 2017). Bardai *et al.* (2016) found this mutation in 5% of the patients, and a very similar frequency was found in the patients from our sample. Mutations in the *IFITM5* gene cause OI type V, as reported in patients with predisposition to develop a hyperplastic callus after fractures or surgical intervention (Semler *et al.*, 2012). However, in our work the clinical team did not identify these changes prior to the genetic study.

The great number of techniques involving high-throughput sequencing becomes a challenge when characterizing the clinical status of variants with uncertain significance (VUS). The characterization of different VUS among distinct groups can aid in the interpretation of the clinical spectrum of rare variants. In the present study, we re-analyzed the following missense and synonymous VUS in the *P3H1*, *CRTAP*, *FKBP10* and *SERPINF1* genes: the *P3H1* c.1812C > T (p.Pro604=) variant; the *P3H1* c.1087A > G (p.Lys363Glu) change; the *CRTAP* c.558A > G (p.Ala186=) change; the *FKBP10* c.590A > G (p.Lys197Arg) missense change; the *FKBP10* c.1546G > A (p.Leu516Phe) variant; the *SERPINF1* c.18A > G (p.Leu6=) synonymous variation and the *SERPINF1* c.21C > A (p.Leu7=) change. These variants were previously reported by Barbirato *et al.* (2015, 2016) using SSCP screening to analyze different causative OI-genes. In our study, we observed that these variations are present in patients who carry pathogenic known mutations in other genes, suggesting that these variants are non-pathogenic changes. Barbirato *et al.* (2015, 2016) failed to detect the pathogenic mutations in patients carrying these variants, probably, because of sensitivity and specificity limitations of the SSCP screening.

The presence of high consanguinity or a founder effect can change the prevalence of a disease among different populations, as described by Bardai *et al.* (2016), who found the majority of mutations to be in the *SERPINF1* and *CRTAP* genes among those who are related with a recessive pattern. Minillo *et al.* (2014) also found the same *SERPINF1* gene mutation in two unrelated Brazilian families, one of them with at least four generations of affected patients and reporting high consanguinity, supporting the hypothesis of a founder effect. In our study, mutations were identified neither in the *SERPINF1* or *CRTAP,* nor in the *PPIB*, *SERPINH1*, *WNT1* or *SP7* genes, suggesting that mutations in these genes are rare in the studied sample.

The identification of mutated genes and the differentiation between dominant and recessive autosomal forms in patients can provide the basis for accurate counseling regarding prognosis and reproductive purposes, allowing the prevention of new cases of the disease in the population. In this study, we analyzed 30 unrelated OI patients for mutations in the 11 most frequently mutated genes in the OI mutations database. Causative changes were found in 97% of the patients. Among these, 47% were in the *COL1A1* gene*,* 30% in the *COL1A2* gene, 10% in the *FKBP10* gene, 7% in the *P3H1* gene and 3% in the *IFITM5* gene. In one patient we found no disease-causing variants. The molecular diagnosis of OI becomes extremely difficult due to the presence of 20 distinct relevant genes*.* Hence, the study of the most frequently mutated genes in OI patients can help to improve molecular strategies of diagnosis for the disease. Our data show that the analysis of these five genes in OI Brazilian patients is able to detect at least 95% of the causative mutations. Nonetheless, distinct populations can have different frequencies of disease-causing variants, and hence, replication of this study in other groups is necessary for knowledge about the profile of OI mutations in distinct communities.

## Supplementary material

The following online material is available for this article

Table S1 Primers used for *COL1A2* gene.

Table S2 Primers used for *SERPINH1* gene.

Table S3 Primers used for *SERPINF1* gene.

Table S4 Primers used for *FKBP10* gene.

Table S5 Primers used for *WNT1* gene.

Table S6 Primers used for *IFITM5* gene.
