# Supplementary material for: Osteogenesis imperfecta in Brazilian patients
Source: Genet Mol Biol. 2019 Aug 15;42(2):344–50. doi: 10.1590/1678-4685-GMB-2018-0043 (PMC6726155; doi:10.1590/1678-4685-GMB-2018-0043)
Supplement: Supplementary file 1 [file 1415-4757-GMB-1678-4685-GMB-2018-0043-suppl1.pdf]

Supplementary Material to: "Osteogenesis imperfecta in Brazilian patients"

Table S1 - Primers used for *COL1A2* gene.

Exon	Amplicon size (bp)	Direction	Sequence (5'-3')
1	228	F	TCTGCGACACAAGGAGTCTG
		R	TGAGAGTCTGCCCTCCAAGT
2	125	F	TGATCCATGAAGTGATACTAATAATTG
		R	CACTGTAGCCAAGGGAATCAA
3	249	F	ACACCAAAATGGAAGCTGTTT
		R	AAACATCAAAGCTACTTTATTTTATGC
4	389	F	TCCAATCCTCCAGCTGAAAA
		R	TCTTCTGCAGTGCATTACCTG
5	385	F	AATTTCCACCCTACTTGCACA
		R	AGGGCTCACAAAGAGAATGG
6	250	F	GCCCTCTTTTAAATAACAACAGAA
		R	AATGGCGTGGTAAAATGTGA
7/8	398	F	CAAACCACAACAATGGCACT
		R	CACCTGCAGGACCCTAAGAA
9	275	F	CCTGGTGAACCTGGTCAAAC
		R	TCAGGCATATTCAGCTTTTGG
10	396	F	CCAAAAGCTGAATATGCCTGA
		R	TGCCTTCGATTCATGTTATGAG
11	332	F	GCAAGTTAATTTGTCACTCTGTGC
		R	TTCTTCCCTTTGGCAAACTC
12	299	F	GACAAGACTTACCCAAGAGAGATT
		R	GGGATTTGAAGTACAGGAAAGAAA
13	300	F	TCTGGCATAATTGAAAAACAATC
		R	AAGAATACAATGCTGAAGGATACA
14/15	297	F	GCAAATGATGCCTGTGACTTT
		R	TCCTTTCAAACTCCCCAGTG

Exon	Amplicon size (bp)	Direction	Sequence (5'-3')
16	322	F	CAACAGACTGGTTGTCAGTTTTT
		R	TTGAAGGGAAGGAATAATTAAGG
17	315	F	TTGCAATTTTGAAGTTTTATGAAGA
		R	TGGAATATTTCGGTTCTTCTTTG
18	249	F	CAAAGGTGGAGTATGGGGAAG
		R	AATGCAGTGTGGTCCATTAGG
19	317	F	GCGTAGCTAAAATGTGCTGCT
		R	TGCTAAGGAAGAGAAAAATAGGAA
20	294	F	TTGAGCTTCTCTTTACCTTGACC
		R	CCAGCAGAGCCCTGTTTAAT
21	377	F	GTAACAAGGGTGAGCCCGTA
		R	AGGCAGATGGAAAGCAGATG
22	296	F	TTTGGCTTGGTTTGTGTCTG
		R	AGGAGGGCCCTATTCAAAAA
23	237	F	CTAGAAAGGGGCTTGCTGCT
		R	AGGAAGTAATGCCAGGTGTG
24	400	F	TTCAGAACTCTTTTCACACTTCC
		R	CAGTCCTGTATTCATTTGGACTC
25	281	F	ATCCGTGGCAGCATCATAA
		R	GGAAACTCCCTGAGACTGGAC
26	238	F	AATCACCGTGGTTAATTTGACA
		R	GGGGATGCCATCTTGAAAAG
27	276	F	AGTATTTGGGCTTTCGTGGGAACC
		R	AAATGGAGATGGCCAGTTTGAGGAC
28	194	F	ACAACAGTGGTGACATACGTTGCT
		R	TGTGGTGGAGAAGAGAGGTACGGT
29	300	F	AGCCACCACCCCCAAACTCA
		R	GGCTCATTCTCTCCATCAGCACCA
30	273	F	TGCACTCATGTAGATACTGCCAGGT

Exon	Amplicon size (bp)	Direction	Sequence (5’-3’)
		R	AGCATCAGAGACTTGTTGCAGGGT
31	350	F	TGCAAACCAGGGCTCGGAAGC
		R	TCGGATTGCTGTTTACTGAGAGGGA
32	300	F	TCAAAGCAGGCAAGAAGCCTGT
		R	GTGAAAACTTGGGCATCCTTGTGC
33	265	F	TGACTGAAGGTATCATAGCATC
		R	GATAAATAAAGTGTGTGTAGTTCT
34	265	F	CCAACCAGAGTGCAGTGAAAGTGT
		R	GCCATAAAAATGAATTGCTGGGGCTC
35/36	371	F	CCACCACTGTTCTCTCTCCCTCCC
		R	ACAGCTCTGGTATTCCGACCCACT
37	334	F	AGTGGGTCGGAATACCAGAGCTGT
		R	TCCCTGTTGCATAGCAGGCACT
38	373	F	AGAGATGCGGGAATGATCCACTTG
		R	TGCCTAGGGCCCACCTTGGTT
39	249	F	ACTCCTTGGTCTATTCCTGGTCACAT
		R	GTTTCTTACCAGTTTCCTATCAGAAGCCA
40	312	F	GAATCTTTGCTGCTCTCTCCAGGC
		R	GCCACTTGAAGATTTGTGAGGGGCT
41	298	F	GGAGGTCATTAGCCTTTTTCTAAGCTGAA
		R	TCTGTCACATTTGAAGTGGCAGCTTT
42	322	F	CCTGAGTAGGGTTGTTTTGGAGGGG
		R	GGTGTAGATACTATGGAGAAGCTGACCA
43	232	F	AGGGTTCGTTACTGAGCACTGGA
		R	AGCTTAGCATCAATCTGGGTTGCATT
44	262	F	AGTGATTAAAATGCAACCCAGATTGATGC
		R	AAGATACCCCTCCCCACTCCTCCAC
45	289	F	CCCCACACTTGGGGATGGTGGA
		R	CAAGTTATTTGTATCAATTCTCAGCATGGACTG

Exon	Amplicon size (bp)	Direction	Sequence (5'-3')
46	331	F	GCAGTATTTGTGGTGAAGTGAGTGCC
		R	ACCTCCAAGAGTGAGATGGAGTTAGCC
47	222	F	CCGGAGTCCATTTAACTAAAGTTTCCCA
		R	GGCTCACTGCTCGCTTTAGCCTCTA
48	279	F	CTAAAGCGAGCAGTGAGCCCCAGG
		R	TGGGTTGTCAAAGTTGTCTTGGTTTAGTC
49	380	F	TGCTGCCATGGATGTCTCCACTGT
		R	TGAAAAGCTCAACTTGTGAGAAGGGTCA
50	434	F	TGGGGTAGACAATCAAAAATGTTACTTATGAGAG
		R	TGGAACCCAGGAAAGGAACAGGTCT
51	319	F	TGGATCTGAGTCTACTCTTCCTGAGATCTTT
		R	TGCCCTCAGCAACAAGTTCAACATCA
52a	359	F	TGACACATGCCAAACAGTGGTTCTT
		R	TGTGCAGAAGAAATGGAAGGATTCAGC
52b	379	F	GCCCAGTCTGTTTCAAATAAATGAACTCAATC
		R	TGGGTTTTAAACTTCCCTCTGTGGAAGA
52c	370	F	ACATTTGCACCACTTGTGGCTTTTG
		R	TGCTGAATCTGAAGAAGATTTGGGCA
