# Supplementary material for: Osteogenesis imperfecta in Brazilian patients
Source: Genet Mol Biol. 2019 Aug 15;42(2):344–50. doi: 10.1590/1678-4685-GMB-2018-0043 (PMC6726155; doi:10.1590/1678-4685-GMB-2018-0043)
Supplement: Supplementary file 2 [file 1415-4757-GMB-1678-4685-GMB-2018-0043-suppl2.pdf]

# Supplementary Material to: "Osteogenesis imperfecta in Brazilian patients"

**Table S2** - Primers used for *SERPINH1* gene.

| Exon | Amplicon size (bp) | Direction | Sequence (5'-3') |
|---|---|---|---|
| 1 | 400 | F | TCGCACTCTGAAGGACACGCT |
| | | R | CGCACCCCCAGATTGCTCGG |
| 2a | 299 | F | AGCTGAGGGTGGTTGTTGGGG |
| | | R | TCTCCACTGCCTGGTCCTTGG |
| 2b | 300 | F | CGGCCTGGCCTTCAGCTTGT |
| | | R | CGCACGAAGTCATCAGCGAAGC |
| 2c | 300 | F | CTGGAAGCTGGGCAGCCGAC |
| | | R | ACTCTTGCAGGGGGTCCTGG |
| 3 | 249 | F | GGGGTGGCTGTGGGCTGTGA |
| | | R | TGTGTGCAGGGGTGGGTCAGA |
| 4 | 397 | F | GTGGGTGGGGGTCCAAGGGT |
| | | R | ACCTGCTGAATTGGTGAGTGGGT |
| 5a | 317 | F | TGGGGTGGAGGGTTTGAGGGT |
| | | R | GGGAGCCGCTTTGGGTGTCC |
| 5b | 293 | F | GCGCAGCCCCAAGCTGTTCT |
| | | R | TGGGAAGGTCCGCTCAGGCA |
| 5c | 369 | F | ACTCCATGGGGTGGGGGTGG |
| | | R | TTCTGGGGGTGCCTGGTCCC |
| 5d | 386 | F | TCAGCTGCCTCCCCAGCTCT |
| | | R | AGGAGACCTTGTCCCCTGGGAAG |
