# Supplementary material for: Osteogenesis imperfecta in Brazilian patients
Source: Genet Mol Biol. 2019 Aug 15;42(2):344–50. doi: 10.1590/1678-4685-GMB-2018-0043 (PMC6726155; doi:10.1590/1678-4685-GMB-2018-0043)
Supplement: Supplementary file 3 [file 1415-4757-GMB-1678-4685-GMB-2018-0043-suppl3.pdf]

Supplementary Material to: "Osteogenesis imperfecta in Brazilian patients"

Table S3 - Primers used for *SERPINF1* gene.

Exon	Amplicon size (bp)	Direction	Sequence (5'-3')
1	369	F	GCCCGGCCCCTGAAGGAAAA
		R	CCTGCCTCTCCCCTGTCCCC
2	299	F	CTGCCCAACCCCTGGGTCCT
		R	CAAGCCTGGCCTGGAACCTG
3	384	F	ACAGCCCCAAGGGGCCAGAA
		R	TCAGCCACGTTTACGCAGAGGC
4	347	F	TGTCTGTGTTCTGGGAGGGGGC
		R	CCAGCCTGGGCAACAGAGCG
5	399	F	ACCAGAACCCGAGCCTGGCA
		R	AGATCGCACCACTGCACTCCA
6	291	F	TCTGGGGACACAGCATGGCG
		R	ATTCCCACTACCCTGTTTTGCTTCTC
7	399	F	CACGGGAGAGGGAAGGCAGC
		R	GCCTGTGGAGCCCTTGCGTT
8a	353	F	ACAGTGCTGCGCCATCCCAG
		R	GGCCCCTGGGGTCCAGAATC
8b	293	F	TTCGTACTGAGGGACACAGACACA
		R	ACCAGGCTAGAAGTAGAGGACCACCA
