# Supplementary material for: Osteogenesis imperfecta in Brazilian patients
Source: Genet Mol Biol. 2019 Aug 15;42(2):344–50. doi: 10.1590/1678-4685-GMB-2018-0043 (PMC6726155; doi:10.1590/1678-4685-GMB-2018-0043)
Supplement: Supplementary file 4 [file 1415-4757-GMB-1678-4685-GMB-2018-0043-suppl4.pdf]

Supplementary Material to: "Osteogenesis imperfecta in Brazilian patients"

Table S4 - Primers used for *FKBP10* gene.

Exon	Amplicon size (bp)	Direction	Sequence (5'-3')
1	400	F	ACCAGCCAGGGCGGGGGT
		R	AATCCGGGGGCGCCCAACC
2	285	F	TGCATCTGTGCCACCATGGGC
		R	ACCCCAGGATCCCTGATTCTGCC
3	376	F	GGTGCTGGGATGAGAGGAAGGGG
		R	TGGAGGTGGGAGAGGCAGGC
4	285	F	GCATGGGGAGCGGGAATCCG
		R	ACCTGAGCAACTGTATGTGGAGGC
5	397	F	GCTGATGGGCGGGAAAGGGC
		R	AGGCAGCTCAGAGTGACCCCC
6	300	F	CTGCCTGGAAGGGGAGGGC
		R	GCGGTGCCTGGAATCCCACT
7	392	F	CCTCAGGGTCGGGAAGGGGT
		R	AGAGGGAGGAGCAGGCGTCG
8	386	F	CTGGGCCCACCTCAGAGGGA
		R	ACTCGGGAGGCTGAGGCAGG
9	337	F	AACTGGCCTGTGGGCTGGGA
		R	CCGGACGGGGGACGTCTTCT
10a	393	F	CCATGACCCTCACTGCCCGC
		R	AGGGTGGTGGGAACACCAGAGA
10b	394	F	GGATGAGGTCCAGGAGCCAACT
		R	CTGCCCTCCAGGAGCCTTCCC
10c	395	F	TGACTGGCTCCTAGGGAAGGGGA
		R	CCCCCTGCCCCTCAACCTGG
