# Supplementary material for: Osteogenesis imperfecta in Brazilian patients
Source: Genet Mol Biol. 2019 Aug 15;42(2):344–50. doi: 10.1590/1678-4685-GMB-2018-0043 (PMC6726155; doi:10.1590/1678-4685-GMB-2018-0043)
Supplement: Supplementary file 5 [file 1415-4757-GMB-1678-4685-GMB-2018-0043-suppl5.pdf]

Supplementary Material to: "Osteogenesis imperfecta in Brazilian patients"

Table S5 - Primers used for *WNT1* gene.

Exon	Amplicon size (bp)	Direction	Sequence (5'-3')
1	493	F	CCATTGTCTGCGCCCCTAA
		R	GGCAGACGGATCCCAGAG
2	477	F	TTCTCTCCAGCCACATACCC
		R	GGTTGATTGTGTGACCCTCC
3	413	F	TTCATGAGGGTGCTGGCC
		R	CCTTATCTCACCGACCGTCA
4a	345	F	CAGTGTCTGGGAGGGTGAC
		R	TGTACGTGCAGAAGTTGGG
4b	355	F	GAAGACCCGGCCCACAAA
		R	TGAGTGCTAGCGAGTCTGTT
4c	424	F	TGCACGAGTGTCTGTGAGG
		R	CGGAGAAAACGCAGGACAAA
4d	344	F	GTCATTCTGCCTGCTCCATC
		R	ACACACTGATGAGGAGGCAG
4e	450	F	GTCGTGGAGCCATTGAACAG
		R	AAAGGGGAGGATAGGGGACA
