# Supplementary material for: Osteogenesis imperfecta in Brazilian patients
Source: Genet Mol Biol. 2019 Aug 15;42(2):344–50. doi: 10.1590/1678-4685-GMB-2018-0043 (PMC6726155; doi:10.1590/1678-4685-GMB-2018-0043)
Supplement: Supplementary file 6 [file 1415-4757-GMB-1678-4685-GMB-2018-0043-suppl6.pdf]

Supplementary Material to: "Osteogenesis imperfecta in Brazilian patients"

Table S6 - Primers used for *IFITM5* gene.

Region	Amplicon size (bp)	Direction	Sequence (5'-3')
5'UTR	309	F	ACAGGGCTATAAGTGAGCGG
		R	TGATGGAGTAGTGGAGCCTC
